# Kikuchi-Fujimoto disease (histiocytic necrotizing lymphadenitis) with atypical encephalitis and painful testitis: a case report

**DOI:** 10.1186/s12883-017-0807-4

**Published:** 2017-02-01

**Authors:** Hidenori Kido, Osamu Kano, Asami Hamai, Hiroyuki Masuda, Yutaka Fuchinoue, Masaaki Nemoto, Chiaki Arai, Teppei Takeda, Fumihito Yamabe, Toshihiro Tai, Mizuki Kasahara, Kenichi Suzuki, Nobuyuki Shiraga, Sota Sadamoto, Megumi Wakayama, Yukitoshi Takahashi, Yasuo Iwasaki, Kazutoshi Shibuya, Yoshihisa Urita

**Affiliations:** 1 Department of General Medicine and Emergency Care, Toho University School of Medicine, 6-11-1 Omorinishi Ota-ku, Tokyo, 143-8541 Japan; 2 Division of Neurology, Department of Internal Medicine, Toho University School of Medicine, Tokyo, Japan; 3 Department of Neurosurgery Radiology, Toho University School of Medicine, Tokyo, Japan; 4 Department of Otorhinolaryngology, Toho University School of Medicine, Tokyo, Japan; 5 Department of Urology, Toho University School of Medicine, Tokyo, Japan; 6 Department of Radiology, Toho University School of Medicine, Tokyo, Japan; 7 Department of Pathology, Toho University School of Medicine, Tokyo, Japan; 8 Epilepsy Center, Shizuoka Institute of Epilepsy and Neurological, Tokyo, Japan

**Keywords:** Encephalitis, Fever of unknown origin, Histiocytic necrotizing lymphadenitis, Kikuchi-Fujimoto disease, Testitis

## Abstract

**Background:**

Kikuchi-Fujimoto disease is a self-limited clinicopathologic entity that is increasingly recognized worldwide. Kikuchi-Fujimoto disease is characterized by cervical lymphadenopathy occurring in young adults. Neurologic involvement is rare, and testitis directly caused by Kikuchi-Fujimoto disease has not yet been reported.

**Case presentation:**

A 19-year-old man was brought to our clinic with complaints of fever, headache, fatigue, and left lower quadrant pain that had persisted for 3 weeks. On physical examination, painful cervical lymphadenopathies were observed. Meningitis was suspected based on a cerebrospinal fluid examination, and left-sided orchitis was diagnosed based on findings from magnetic resonance imaging and ultrasonography. However, neither antibiotics nor antiviral drugs were effective in treating the patient’s symptoms. On the 20^th^ day of hospitalization, the patient experienced a loss of consciousness, and brain T2-weighted magnetic resonance imaging showed asymmetrical, high-signal intensities in both basal nuclei and the left temporal lobe. Encephalitis was suspected, and the patient was treated with intravenous prednisolone pulse therapy (1 g/day) for 3 days and intravenous immunoglobulin therapy for 5 days. A left cervical lymph node biopsy showed apoptotic necrosis in paracortical and cortical areas with an abundance of macrophages and large lymphoid cells, which had irregular nuclei suggestive of Kikuchi-Fujimoto disease; the pathological findings from a brain biopsy were the same as those of the cervical lymph node biopsy. The encephalitis and cervical lymphadenopathies followed a benign course, as did the testitis.

**Conclusions:**

This is the first report of Kikuchi-Fujimoto disease involving painful testitis and pathologically proven asymmetrical brain regions. Kikuchi-Fujimoto disease should be included in the differential diagnosis when a patient presents with encephalitis, testitis, and fever of unknown origin.

## Background

Kikuchi-Fujimoto disease (KFD), or histiocytic necrotizing lymphadenitis, is known to be a benign disease that usually resolves spontaneously. It is a rare disease and was first reported in Japan in 1972 [1, 2]. KFD usually affects female patients under the age of 30 years and is characterized by regional lymphadenopathy with tenderness, predominantly in the cervical region, and is usually accompanied by mild fever and night sweats. It is of clinical importance because it is often misdiagnosed as tuberculosis, lymphoma, or systemic lupus erythematosus (SLE); therefore, the diagnosis should be confirmed by biopsy study. Diseases that generally accompany KFD are SLE, arthritis, mixed connective tissue disease, and similar conditions; however, comorbid testitis has not yet been reported. Neurological complications occur in about 11% of cases, as revealed by a review of 244 cases in which a minority of cases evolved to developing encephalitis [3]. Brain magnetic resonance imaging (MRI) includes symmetrical hyperintense T2-weighted and FLAIR (fluid-attenuated inversion recovery) signals in the temporal lobes, pons, and periaqueductal gray matter [4–7]. This is the first report of KFD occurring with testitis and pathologically proven asymmetrical brain regions.

## Case presentation

**Fig. 1:**
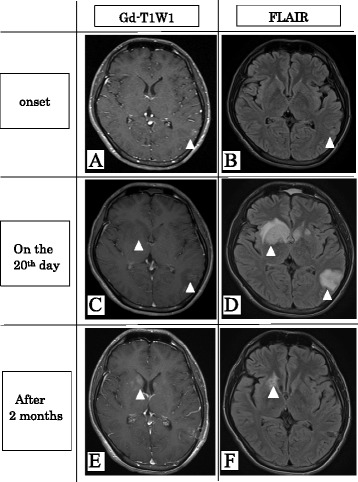
Axial T1-weighted MRI with gadolinium contrast (**a**) and FLAIR-weighted MRI (**b**) show mild enhancement and a high-intensity area, respectively, in the left temporal lobe at onset (*arrow heads*). Following periods of acute exacerbation on the 20th day, axial T1-weighted MRI with gadolinium contrast (**c**) showed slightly larger enhancement, and FLAIR-weighted MRI (**d**) revealed an asymmetrical hyperintense signal in the left temporal lobe and both basal nuclei. After 2 months of hospitalization, the abnormal lesions became smaller (**e**, **f**)

**Fig. 2:**
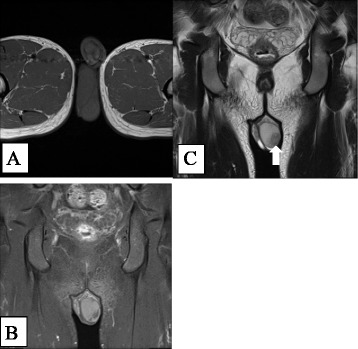
**a** Axial T1-weighted magnetic resonance image shows that the lesion has an isointense signal. **b** After administration of gadolinium contrast, material internal enhancement is not observed. **c** Coronal T2-weighted magnetic resonance image shows that the lesion has low signal intensity (*arrow*)

**Fig. 3:**
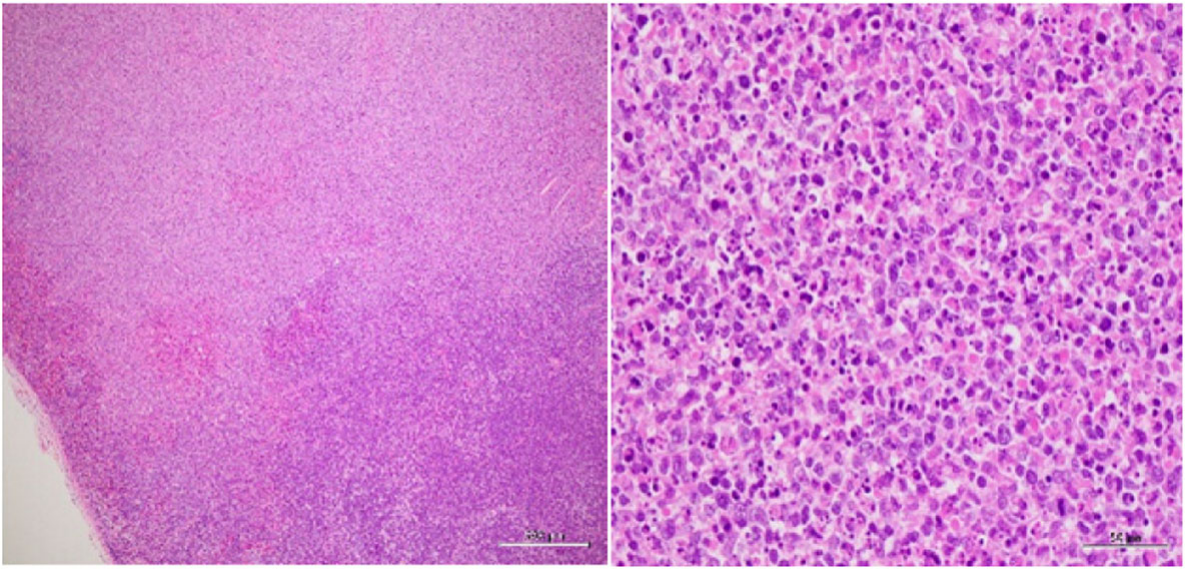
Left cervical lymph node biopsy shows conspicuous apoptotic necrosis in paracortical and cortical areas with an abundance of macrophages and large lymphoid cells, which have irregular nuclei [hematoxylin-eosin staining × 4 (*left*), ×40 (*right*)]

**Fig. 4:**
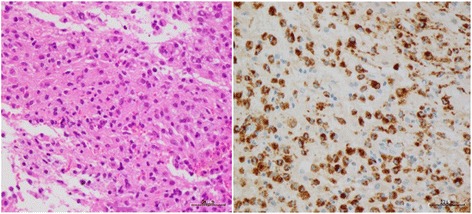
Brain biopsy shows an abundance of CD68+ macrophages and histiocytes, as in the cervical lymph node. [hematoxylin-eosin × 40 (*left*), anti-CD68 (*right*)]

A 19-year-old man presented to our hospital with complaints of fever (39.5 °C), headache, fatigue, night sweats, and lower abdominal pain, which had been present for about 1 month. For 20 days, he had used anti-flatulent medications before presenting to our hospital. He had no history of close contact with pets, he did not travel often, and similar complaints were not present in his social circle. According to the patient’s physical examination on admission, his general health status was good; body temperature, pulse, and blood pressure were 37.2 °C, 102 beats/min, and 106/70 mmHg, respectively. He was alert, oriented, and cooperative. Mobile, painful lymphadenopathies were observed on both sides of the cervical region, of which the largest was 2 × 1.6 cm in size. Bilateral cervical lymphadenopathy was present and involved a posterior group of cervical lymph nodes. Cardiac, respiratory, and neurological examination revealed no abnormality; vegetation was not detected by transthoracic echocardiography. Laboratory investigations showed leukopenia (total leucocyte count: 3200 cells/mm^3^). Cerebrospinal fluid (CSF) analyses were normal. No positive results that could explain the high body temperature were found during serological testing for Epstein Barr virus (EBV); cytomegalovirus (CMV); hepatitis A, B, and C; parvovirus; human herpesvirus; chlamydia trachomatis; mumps; human immunodeficiency virus; echo virus; coxsackie virus enterovirus; and measles spp. Results of tests for rheumatologic markers (ferritin, anti-nuclear antibody, anti-neutrophil cytoplasmic antibody, anti-cyclic citrullinated peptide antibodies, extractable nuclear antigen antibody panel, anti-double stranded DNA, and rheumatoid factor) were also negative. Results of tuberculosis polymerase chain reaction (PCR) and herpes simplex PCR were also negative in the CSF. Blood and CSF cultures showed no growth after incubation for 6 to 8 weeks. Brain T2-weighted and FLAIR MRI sequences showed a slightly hyperintense signal of about 4 mm in the left temporal lobe (Fig. 1), and antibiotics and acyclovir treatment were begun because we could not rule out encephalitis. However, the patient’s body temperature could not be reduced by routine anti-inflammatory drugs and intravenous paracetamol. Thoracic and abdominal computed tomography (CT) and MRI scans revealed a testicular lesion having no contrast effect (Fig. 2); lymph nodes were predominantly located on the right side of the cervical region, with the largest measuring 2 cm in diameter. Thus, one of the lymph nodes located in the right cervical region was removed and sent to pathology for analysis. On the 4th day of hospitalization, an excision biopsy of the cervical lymph node was performed. Microscopic examination showed that the lymph nodes were expressed in paracortical and cortical areas of conspicuous apoptotic necrosis with an abundance of enlarged lymphocytes, which had prominent nucleoli in the absence of neutrophils and a proliferation of macrophages (Fig. 3). Results of tests using in situ hybridization to detect EBV with marked paracortical expansion due to a proliferation of immunoblasts were negative. On the 20th day of hospitalization, the patient’s general status suddenly and temporarily worsened. The signal intensity on brain MRI had changed and showed an asymmetrical hyperintense signal in the wider larger left temporal lobe and both basal nuclei (Fig. 1) with slight Gd-enhancement. CSF protein levels and pleocytosis were increased (protein: 50 mg/dL; pleocytosis: 24 cells/μL), but CSF cultures were negative, and cytology showed no malignant findings. Anti-NMDA, CO, Tr, GAD65, Zic4, Titin, SOX1, Rec, Hu, Yo, Ri, Ma-2/Ta, Cv2, and Amp antibodies associated with auto-immune-mediated encephalomyelitis were all absent. Oligoclonal bands were also not detected in the CSF sample. Since acute disseminated encephalomyelitis (ADEM) was suspected, the patient was managed with intravenous prednisolone pulse treatment (1 g/day) for 3 days and intravenous immunoglobulin treatment for 5 days, and these therapies were repeated 3 times every 3 weeks. Electroencephalography showed no waves characteristic of epilepsy, but levetiracetam 1000 mg/day was begun. On the 34^th^ day of hospitalization, MRI investigation of the brain revealed that the previous abnormalities were present and had not changed. Since the cervical lymph node biopsy before steroid therapy did not reveal lymphoma, a brain biopsy of the temporal lobe was performed. Steroids can obscure diffuse large B-cell lymphoma or intravascular lymphomatosis; however, no recurrence was experienced. The pathological results revealed many CD68+ macrophages and histiocytes, as in the cervical lymph node, suggestive of KFD (Fig. 4). The cervical lymphadenopathies disappeared after 2 months of hospitalization, and the previous abnormalities on MRI became smaller during follow-up. The testicular mass that showed a contrast effect on MRI and CT similarly was smaller (Fig. 1); therefore, we suspected that the testitis had been caused by KFD. His complaints of fever, headache, and fatigue gradually improved; he was discharged on the 90th day of hospitalization. He was followed for more than 3 months in our clinic and did not show any recurrence.

## Discussion and Conclusions

KFD was first described in Japan in 1972 and has increasingly been recognized worldwide since 1982 [8]. According to a review [3] that summarized 244 KFD cases, the mean age was 25 (1–64) years, and 70% were younger than 30 years; 33% were men, and 77% were women. Most of the cases were reported from East Asia and the Far East (50%), and the others were from Europe (27%) and America (7%). As in the present case, the most common symptoms were fever (35%), fatigue (7%), and cervical lymphadenomegaly (100%) [3]. Our case did not show any evidence of SLE, lymphoma, tuberculosis, arthritis, or viral disease based on laboratory findings and cervical lymph node biopsy.

Testitis has not been reported as a co-morbid disease in KFD. After diagnosing KFD based on results of the cervical lymph node biopsy, we could not explain the central nervous system (CNS) involvement in this case. Neurological involvement, including aseptic meningitis and encephalitis, is rare, and abnormal MRI findings in KFD usually show symmetrical, hyperintense T2 and FLAIR signals in the temporal lobes, pons, and periaqueductal gray matter [4–7]. Therefore, we considered the possibility of co-morbid ADEM. Avkan-Oguz et al. [6] reported the co-existence of KFD and ADEM based on brain MRI findings without a brain biopsy. Our case did not show symmetrical lesions, which were reported in the previous KFD reports with CNS involvement; hence, we evaluated brain pathology, and the results were consistent with those for KFD and showed a benign course. Although the etiology of the disease remains unknown, infectious or autoimmune pathogenesis is thought to be responsible. The factors that likely result in the infection include herpes simplex virus, EBV, CMV, human herpes virus 6, varicella zoster virus, HIV, rubella, measles, coronavirus, coxsackie virus, hepatitis A and B, *Yersinia enterocolitica*, toxoplasma, influenza viruses, mumps, streptococci, leptospira, and chlamydia. It has also been reported that the disease may occur after rabies, diphtheria-tetanus, poliomyelitis, hepatitis B, and influenza vaccines are injected [9]. KFD may initially be mistaken for various benign and malignant diseases, such as tuberculosis, lymphoma, SLE, limbic encephalitis, and sarcoidosis. On the other hand, with regards to clinical and laboratory presentations, KFD frequently mimics SLE, arthritis, mixed connective tissue disease, aseptic meningitis, encephalitis, tuberculous lymphadenitis, malignant lymphoma, and some other benign and/or malignant diseases [3, 10, 11]. In addition to painful cervical lymphadenopathies and aseptic meningitis, our case showed testitis and encephalitis, which are not typical MRI findings compared with those reported in a previous case report. Previous papers reported that most cases of KFD had a benign course; therefore, it was difficult to evaluate the effect of our therapies, such as prednisolone and intravenous immunoglobulin treatment. The definitive method for confirming the diagnosis of KFD is with a lymph node biopsy, and therefore, physicians should not hesitate to perform one in order to diagnose the condition. In addition, if progressive CNS involvement is observed, either steroid therapy or brain biopsy should be considered. Thus, KFD may exist on a wide spectrum of diseases, and we suggest that KFD should be included in the differential diagnosis of lymphadenopathy, encephalitis, testis, and fevers of unknown origin.

In conclusion, this is the first report of KFD involving testitis and pathologically proven asymmetrical brain regions.

## Abbreviations

ADEM: Acute disseminated encephalomyelitis
CMV: Cytomegalovirus
CNS: Central nervous system
CSF: Cerebrospinal fluid
EBV: Epstein-Barr virus
FLAIR: Fluid-attenuated inversion recovery
HIV: Human immunodeficiency virus
KFD: Kikuchi-Fujimoto disease
MRI: Magnetic resonance imaging
PCR: Polymerase chain reaction
SLE: Systemic lupus erythematosus
